# Chicken MBD4 Regulates Immunoglobulin Diversification by Somatic Hypermutation

**DOI:** 10.3389/fimmu.2019.02540

**Published:** 2019-11-01

**Authors:** Ryan Costello, Jose F. Cantillo, Amy L. Kenter

**Affiliations:** Department of Microbiology and Immunology, University of Illinois at Chicago, Chicago, IL, United States

**Keywords:** Ig, somatic hypermutation, B cells, uracil DNA glycosylase, DT40, CRISPR

## Abstract

Immunoglobulin (Ig) diversification occurs via somatic hypermutation (SHM) and class switch recombination (CSR), and is initiated by activation-induced deaminase (AID), which converts cytosine to uracil. Variable (V) region genes undergo SHM to create amino acid substitutions that produce antibodies with higher affinity for antigen. The conversion of cytosine to uracil in DNA promotes mutagenesis. Two distinct DNA repair mechanisms regulate uracil processing in Ig genes. The first involves base removal by the uracil DNA glycosylase (UNG), and the second detects uracil via the mismatch repair (MMR) complex. Methyl binding domain protein 4 (MBD4) is a uracil glycosylase and an intriguing candidate for involvement in somatic hypermutation because of its interaction with the MMR MutL homolog 1 (MLH1). We found that the DNA uracil glycosylase domain of MBD4 is highly conserved among mammals, birds, shark, and insects. Conservation of the human and chicken MBD4 uracil glycosylase domain structure is striking. Here we examined the function of MBD4 in chicken DT40 B cells which undergo constitutive SHM. We constructed structural variants of MBD4 DT40 cells using CRISPR/Cas9 genome editing. Disruption of the MBD4 uracil glycosylase catalytic region increased SHM frequency in IgM loss assays. We propose that MBD4 plays a role in SHM.

## Introduction

Activation induced deaminase (AID) is essential for both Ig somatic hypermutation (SHM) and class switch recombination (CSR) in mature B cells (1, 2). SHM increases diversification of V region genes and when followed by selection, results in improved antibody:antigen binding affinities (1). CSR is responsible for diversification of Ig effector functions (3, 4). AID initiates SHM and CSR by deaminating deoxycytidine (dC) to deoxyuracil (dU) that is processed by base excision repair (BER) and mismatch repair (MMR) [reviewed in (4–6)]. The BER pathway facilitates the excision of dU bases by a uracil DNA glycosylase (UNG) leaving an abasic site that is cleaved by apurinic/apyrimidinic endonuclease (APE) producing a single strand break. The mismatch repair (MMR) pathway also functions to detect and excise mismatches generated by DNA replication and AID mediated deamination and form ssDNA nicks and gaps (6). During SHM the MMR pathway is co-opted to promote the re-synthesis of the nicks and gaps using error prone polymerases Pols η and ζ resulting in nucleotide substitutions (7).

There are four uracil DNA glycosylases, that are capable of recognizing and removing dU in U:G mismatches, UNG, SMUG1, TDG, and methyl binding domain protein 4 (MBD4) (8, 9). Genetic studies show that UNG deficiency in mice (10) and humans (11) reduces CSR 90–95% and perturbs SHM mutation spectra but does not alter SHM frequency. SMUG1 plays little natural role in CSR since it is poorly expressed in activated B cells (12, 13). TDG cannot substitute for UNG during CSR in activated B cells (5). A recent study suggests that during SHM TDG and SMUG1 provide uracil glycosylase activity in the absence of UNG (14).

We were intrigued by the association of MBD4 and AID in zebrafish (15) and its link to MMR through its interaction with MutL homolog 1 (MLH1) (16, 17). However, we and others found no role for MBD4 in CSR or SHM when Mbd4 5′ exons were deleted in mice (18, 19). In contrast, deletion of Mbd4 3′ exons have striking consequences for CSR in the CH12 lymphoma cell line (20). Our studies identified two isoforms of Mbd4 transcripts, the canonical long form and a new short isoform of Mbd4 that is retained in mice with 5′-Mbd4 deletions and may support uracil glycosylase activity (19, 20). The interaction between MBD4 and MLH1 has been postulated to play a role in the coordination of BER and MMR to rectify T:G and U:G mismatches (21). MBD4-MLH1 interaction has been confirmed in activated splenic B cells using co-immunoprecipitation studies (19). Interestingly, ~43% of primary human colorectal carcinomas with- and without- microsatellite instability (MSI) also harbor inactivating mutations in Mbd4 (22–24). Examination of Mbd4 in these tumors revealed mutations resulting in a predicted truncated protein lacking the C-terminal glycosylase domain and are similar to the 3′Mbd4 deletions we engineered into the CH12 clones. However, it was not possible to study SHM in the CH12 cell line.

Here, we explore the contribution of MBD4 to SHM in chicken B cells. To begin, MBD4 is highly conserved in mammals, birds and insects. The predicted three-dimensional structure of the chicken MBD4 uracil glycosylase domain is essentially identical with that of human making it highly likely that it has functional activity. To test MBD4 in SHM we used the well-established chicken DT40 B cell line that has been engineered to allow only SHM to diversify V exons (25, 26). We constructed DT40 sub-lines in which segments of the Mbd4 3′ uracil glycosylase domain were deleted using CRISPR/Cas9 genome editing. Loss of the highly conserved amino acids in the uracil glycosylase domain led to increase of SHM frequency in the DT40 deletion variants as compared to control cells. Our studies provide the first evidence of a role for MBD4 in SHM.

## Methods

### MBD4 Structural Analysis

Using the human MBD4 aa sequence as the control, annotated MBD4 proteins from chicken, mouse, shark, platypus, coelacanth and aphid were identified using Uniprot. Multiple amino acid sequence alignments and the estimated homology to the human MBD4 protein were performed using Clustal Omega's multiple sequence alignment tool (https://www.ebi.ac.uk/Tools/msa/clustalo/). The human MBD4 glycosylase domain crystal structure was previously solved and was used here as a reference structure for comparison (27). The predicted three-dimensional (3D) structure of the chicken MBD4 glycosylase domain was derived using the SWISSMODEL workspace via the ExPASy web server (https://swissmodel.expasy.org/interactive) using the PDB template 4E9E human MBD4 glycosylase (27). The structural superimposition of human and chicken MBD4 glycosylase domains was performed using PyMOL. Comparison of the chicken MBD4 and predicted MBD4 in CRISPR/Cas9 edited DT40 clones were derived using the same strategy.

### DT40 Cells and Cell Culture

The DT40 control cells were AID^R^ψV^−^ (28). All DT40 cells were maintained in culture at 39.5°C with 5% CO_2_ in RPMI 1640 (Corning) supplemented with 10% fetal bovine serum (Atlanta Biologicals) 2% Penicillin Streptomycin (Gibco), 1% L-glutamine (Gibco), 1% chicken serum (Sigma), and 0.1% β-mercaptoethanol (Sigma).

### Generation of MBD4^Δ/Δ^ Cells

The chicken Mbd4 gene in DT40 cells was disrupted in exon 5 at genomic coordinates chr12:19,878,301-19,881,713 (build GRCg6a). Target sequence (5′CTGCACGGAATCGGAAAGTA-3′) for CRISPR/Cas9 editing was identified using www.crispr.mit.edu (no longer available). DT40 cells were CRISPR/Cas9 edited using two strategies. SgRNA-CRISPR/Cas9 expression plasmids were constructed by cloning DNA oligonucleotides complementary to the Mbd4 gRNA (5′CUGCACGGAAUCGGAAAGUA-3′) (IDT) into pX330 (29) (pX330-U6-Chimeric_BB-CBh-hSpCas9; Addgene plasmid #42230) as described (30). PX330 plasmids were transformed into DH5α (Thermo fisher Scientific) and cloned inserts were validated by DNA sequencing using primer hU6-F: 5′-GAGGGCCTATTTCCCATGATT-3′. For ribonuclear protein (RNP) based delivery, Alt-R S.p. Cas9 Nuclease V3 (1 μM) (IDT) was mixed with gRNA (2 μM) comprised of Alt-R CRISPR-Cas9 crRNA (IDT) and Alt-R CRISPR-Cas9 tracrRNA conjugated to ATTO 550 dye (IDT) according to manufacturer's instructions. Nucleofections were carried out when DT40 cells were 80-100% confluent using Amaxa Cell line nucleofector Kit T (Lonza) and program B-023 following the manufacturer's instructions. Cells were allowed to recover for 24 h, stained with anti-chicken IgM-PE (Southern Biotech) and then FACS (MoFlo Astrios) sorted for IgM^+^GFP^+^ cells. Cells nucleofected with RNP were sorted for IgM^+^GFP^+^Atto550^+^. Cells were submitted to limiting dilution to isolate subclones. Subclones were expanded for 10–14 days and genomic DNA was harvested using the alkaline lysis method. Indels in Mbd4 exon 5 were identified by size change of the PCR amplification product using primers F1: 5′-CAGTCCTGGTGGTTGGTTTT-3′ and R1: 5′-TGAGGCAGACTTGCAGAAGA-3′ and verified by DNA sequence analysis using the same primers. MBD4^Δ/Δ^.14 clone was generated via the RNP method and MBD4^Δ/Δ^.11 and MBD4^Δ/Δ^.12 were generated via the plasmid-based method.

### RT-PCR

Total RNA was extracted from DT40 (3 × 10^6^) cells using Trizol as recommended by the manufacturer. RNA (1 μg) was pretreated with DNase I (Invitrogen) and then cDNA was synthesized using Superscript II (Invitrogen). Quantitative (q) RT-PCR was performed for 18S RNA using SYBRGREEN (Life Technologies), as described (31) with 18S F: 5′-TTGACGGAAGGGCACCACCAG-3′ and 18S R: 5′-GCACCACCACCCACGGAATCG-3′ primers. Semi-quantitative PCR amplification of Mbd4 exons 5-6 was performed using DreamTaq Polymerase (Thermo Fisher Scientific) and 26-32 PCR cycles with primers F2: 5′-TCTTCTGCGTCAATGAATGG-3′ and R2: 5′-GTCCACGCTCAGCTTCTCAT-3′. Thermal cycler conditions: 95°C initial denaturation for 2 min 30 followed by cycles of 30 s at 95°C denaturation, 30 s at 61°C annealing, 20 s extension at 72°C.

### SHM of the DT40 IgM Locus Assessed in the IgM Loss Assay

DT40 cells from each genotype were FACS sorted (MoFlo Astrios) for IgM^+^GFP^+^ cells on day 0. IgM^+^GFP^+^ cells were subcloned by limiting dilution and 24 parental subclones and 12 subclones each from MBD4^Δ/Δ^.14, MBD4^Δ/Δ^.11 and MBD4^Δ/Δ^.12, were obtained. All subclones were maintained in culture for 28 days then re-analyzed (BD LSR Fortessa) for surface IgM and GFP.

### Mutation Analysis of the IgL V Region in DT40^c^ and Mbd4 Deletion Clones

Subclones (*n* = 12) from the control and MBD4^Δ/Δ^.14, MBD4^Δ/Δ^.11 and MBD4^Δ/Δ^.12 cell lines that were isolated in the IgM loss assay were taken for mutation analysis. The IgL V region was PCR amplified with IgL F 5′ TTCTCCCCTCTCTCCTCTCC 3′ and IgL R 5′AGACGAGGTCAGCGACTCA 3′ primers using Q5 High-Fidelity DNA Polymerase (New England BioLabs, M0491S) and an amplification protocol of 35 cycles at 55 sec/98C, 20 sec/62C, 15 sec/72C with a final extension time of 2 min/72C. Amplicons were 390 bp and were gel purified and submitted to Sanger sequencing on both the forward and reverse strands. Mutations were scored when found on both the forward and reverse strands. The reference DNA sequence was previously described (28) and matched the DNA sequence from unmutated DT40c cells used in our studies. No insertions or deletions were detected.

### Statistical Analyses

Statistical analyses were performed using non-parametric Kruskal-Wallis test and GraphPad Prism (GraphPad Software, La Jolla California USA).

## Results

### The MBD4 Glycosylase Domain Is Highly Conserved Amongst Animal Species

**Figure 1:**
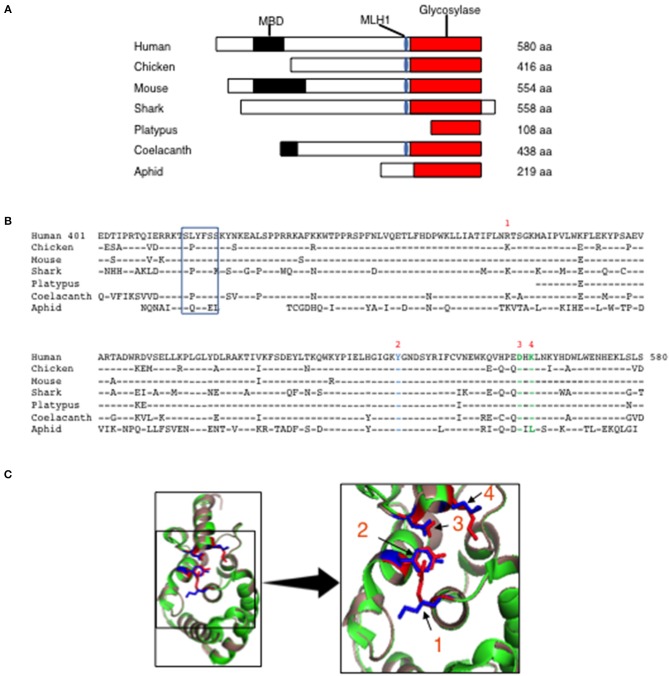
Evolutionary conservation of MBD4 glycosylase domain. **(A)** Schematic (drawn to scale) of MBD4 protein in human (*Homo sapiens*), chicken (*Gallus gallus*), mouse (*Mus musculus*), ghost shark (*Callorhinchus milii*), platypus (*Ornithorhynchus anatinus*), ocean coelacanth (*Latimeria chalumnae*), and green aphid (*Schizaphis graminum*). The methyl binding domain (MBD) (black box), MLH1 binding domain (blue oval) and glycosylase domain (red box) are indicated as appropriate and amino acid (aa) number is listed. **(B)** The annotated human glycosylase domain exons 4–8 (aa 401–580) was used as the original sequence. Multiple aa alignments were performed showing conservation with chicken aa 237-416 (94.4%), mouse aa 375–554 (93.3%), ghost shark aa 347–528 (87.2%), platypus aa 1–108 (92.6%), ocean coelacanth aa 259–438 (90.6%) and green aphid aa 59–219 (70.4%). Conserved aa are indicated by dashes and non-conserved aa are noted using the single letter aa code. The MLH1 binding domain is boxed and the predicted catalytic amino acids in the glycosylase domain are numbered R368 (1), Y540 (2), D560 (3) and K562 (4) using the human MBD4 aa numbering system. **(C)** (Left panel) The three-dimensional (3D) crystal structure of MBD4 glycosylase domain from human (green) (27) and the predicted chicken (dark salmon) structure were aligned using PyMOL with catalytic amino acids shown in red (human) and blue (chicken). (Right panel) Comparison of the crystal structure involving the catalytic aa, R368 (1), Y540 (2), D560 (3), and K562 (4) in the human (red) and chicken (blue) glycosylase domains is shown in high magnification.

Human MBD4 has 2 distinct domains, the MBD (aa 82–147) and a uracil glycosylase domain (aa 426–580) that are separated by a linker (aa 401–425) (16). The linker domain contains an MLH1 binding motif (SLYFSS) that may link MBD4 function with the MMR pathway of DNA repair (16). To determine whether the MBD and the uracil glycosylase elements are conserved we interrogated the Uniprot protein database for annotated Mbd4 genes across multiple species and compared them to the human MBD4. The MBD4 uracil glycosylase domain (red rectangle) was detected in mammals (mouse, human, platypus), birds (chicken) fish (shark and coelacanth) and insects (aphid) (Figure 1A). However, the MBD (black rectangle) was only sporadically paired with the MBD4 uracil glycosylase domain (Figure 1A). The MLH1 binding motif (blue oval) was variably conserved in mammals as it was present in human and mouse but absent in platypus (Figure 1A). In all annotated MBD4 uracil glycosylase domains aa sequence homology ranged 70–95% (Figure 1B). A value of 20% protein sequence homology is considered significant (32). Thus, the MBD4 uracil glycosylase domain is highly conserved in animals.

To further assess the conservation of the MBD4 uracil glycosylase domain we analyzed the preservation of the inferred catalytically active R468, Y540, D560, K562 residues found in human (27) across the annotated MBD4 sequences. To begin, R468 (identified as 1) is conserved between humans and mice and a functionally similar amino acid, lysine, is in the synonymous position for all other species (Figure 1B). Y540 (2) and D560 (3) are present in all species studied. K562 (4) is conserved in all species except aphid (insects), in which it is replaced by the chemically distinct leucine (Figure 1B). Thus, the catalytically active residues within the MBD4 uracil glycosylase domain are very highly conserved in chicken as well as other species.

Next, it was important to determine whether other aa differences in the chicken MBD4 impact on the overall structure that in turn could affect function. The DNA binding site of human MBD4 uracil glycosylase is in a cleft formed by the inferred catalytic residues orientated toward the active site (27, 33). The DNA helix is bound to the enzyme via hydrogen bonds formed between Y376 and the mismatched uracil or thymine, which is “flipped” into the active site by bending at a 55° angle, with K398 acting as a dock (27, 33). K304 fills the space left by the flipped-out base and D396 is thought to catalyze the removal from the DNA helix by directly breaking the N-glycosidic bond between the sugar and the mismatched base (27, 33). We aligned the predicted three-dimensional (3D) structure of the chicken MBD4 uracil glycosylase domain with the previously defined human MBD4 crystal structure that had been solved at the 1.8A° resolution (27). Strikingly, the predicted chicken MBD4 uracil glycosylase structure (dark salmon) is almost identical with the human MBD4 crystal structure (green) (Figure 1C). All four catalytically active amino acids, K304 (1), Y376 (2), D396 (3), and K398 (4) in chicken (marked in blue) are similarly aligned with those in human (red) strongly suggesting that MBD4 glycosylase function is preserved in chicken (Figure 1C). This comparative analysis of human and chicken MBD4 crystal structures indicates that the glycosylase domains fold in a similar fashion and identifies critical catalytically active amino acid residues as potential targets in genome editing.

### Deletions in Chicken MBD4 Are Predicted to Disrupt MBD4 Structure and Function

**Figure 2:**
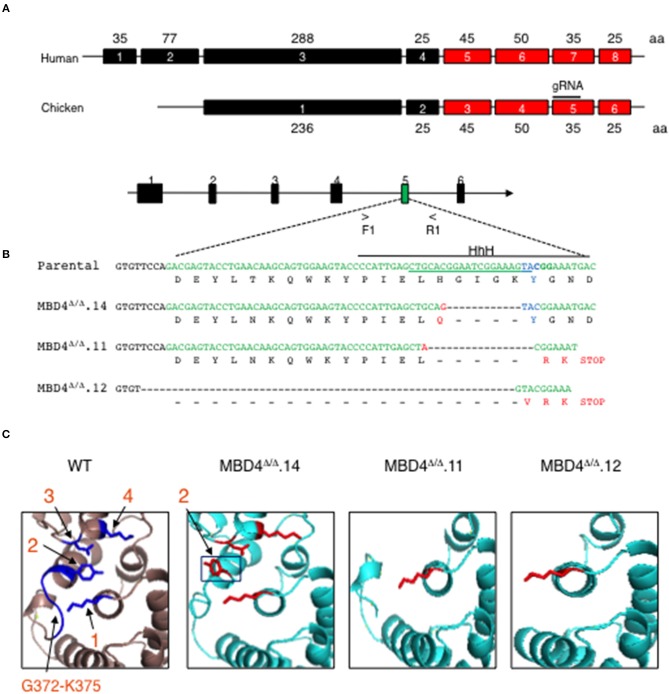
CRISPR/Cas9 genome generated deletions in the chicken MBD4 glycosylase domain of DT40 cells. **(A)** Schematic of human and chicken MBD4 exons (not drawn to scale) Exon numbers are given in boxes with amino acid (aa)s in each exon labeled. The glycosylase domain is highlighted in red and guide (g) RNA in chicken exon 5 annotated. **(B)** Schematic of Mbd4 gene showing exon 5 targeted by CRISPR/Cas9 editing (not drawn to scale). Mbd4 PCR amplification using primers F1 and R1 was used to identify indels. The gRNA sequence is underlined along the parental DNA (upper) strand and catalytic aa Y376 (lower strand) shown in blue. HhH motif is displayed by solid black line. Deletions in the Mbd4 gene for clones Mbd4^Δ/Δ^.14 (12 bp deletion), Mbd4^Δ/Δ^.11 (17 bp deletion), and Mbd4^Δ/Δ^.12 (62 bp deletion), as compared to the parental sequence (aa 357–379) and predicted aa sequences are shown. **(C)** The predicted 3D structure of the chicken MBD4 glycosylase domain (aa262-416) is compared to mutated clones, as indicated. Critical amino acids in WT MBD4 are numbered K304 (1), Y376 (2), D396 (3), and K398 (4). Amino acids G372-K375 are present only in WT.

To assess the involvement of MBD4 in SHM in chicken DT40 cells we generated deletions in the Mbd4 gene that affect the catalytically active amino acid Y376 using CRISPR/Cas9 genome editing (Figure 2A). The Mbd4 exon structure in human (exons 4–8) and chicken (exons 2–6) is essentially identical (Figure 2A). Chicken MBD4 Y376 in exon 5 is synonymous with the human Y540 in exon 7 and deletion of this or surrounding residues leads to perturbation of the catalytic center and its ability to interact with a mismatched base (27) (Figure 2A). To this end, we designed a guide (g) RNA targeting exon 5 in chicken DT40 cells (Figures 2A,B). DT40 AID^R^ψV^−^ B cells used here were previously deleted for endogenous AID and AID expression was complemented by Tg AID linked to GFP (28). Consequently, AID expression can be followed by GFP expression. DT40 AID^R^ψV^−^ cells, hitherto referred to as control DT40 (DT40^c^) were also deleted for pseudo V (ψV) donor genes which abolished gene conversion and enforced SHM at the rearranged light chain VJ segment in IgM (28). Introduction of AID lesions into the VJ exon of DT40^c^ cells may cause frameshifts or missense mutations and lead to loss of Ig expression. Hence, SHM can be tracked in DT40^c^ cells by IgM expression loss over time (28).

In this study genome editing was performed using DT40^c^ cells that were transfected with gRNA and Cas9, then sub-clones were isolated by limiting dilution and screened for Mbd4 exon 5 deletions using PCR. Deletion clones were validated by DNA sequencing (Figure 2B). Three independent CRISPR/Cas9 edited subclones, MBD4^Δ/Δ^.14, MBD4^Δ/Δ^.11 and MBD4^Δ/Δ^.12, were isolated and each contained a unique deletion in exon 5 located adjacent to the gRNA position (Figure 2B). We compared the DNA sequence of the Mbd4 DT40 deletion clones to the intact chicken Mbd4 gene sequence (Figure 2B). The 12 bp deletion in MBD4^Δ/Δ^.14 led to H371Q and loss of four residues including G372-K375 which form part of a helix hairpin helix (HhH) motif adjacent to Y376 (Figure 2B). The MBD4^Δ/Δ^.11 associated 17 bp deletion caused loss of six aa, H371-Y376, followed by a frameshift and a premature stop codon (Figure 2B). The large 62 bp in MBD4^Δ/Δ^.12 begins in intron 4 and caused a frameshift with altered expression of three aa residues and a premature stop codon at the 3′ end of exon 5 (Figure 2B). In all three cases, part of the HhH motif (aa 367–384) was lost which is important for uracil glycosylase activity (27, 33). MBD4^Δ/Δ^.11 and MBD4^Δ/Δ^.12 subclones have also lost the three critical aa; Y376, D398, and K398.

Using the predicted chicken MBD4 3D structure as a reference we analyzed MBD4 structure in MBD4^Δ/Δ^.14, MBD4^Δ/Δ^.11, and MBD4^Δ/Δ^.12 subclones. The chicken aa residues K304, Y376, D396 and K398 are highlighted (blue) and numbered 1–4 (Figure 2C, left panel). MBD4^Δ/Δ^.14 lost aa G372-K375 which likely causes the orientation of Y376 (black box) to swing out of the active site and now face away from the other catalytic amino acids which remain intact. This change in Y376 orientation could lead to diminished uracil glycosylase activity as the mismatched uracil or thymine would not adequately bind into the active site of the enzyme. In MBD4^Δ/Δ^.11, five aa including Y376 in exon 5 and D396 and K398 in the C-terminus are deleted. The loss of Y376 and K398 is predicted to affect DNA binding and a reduction in catalysis of any bound DNA. In MBD4^Δ/Δ^.12, nineteen aa were deleted including the G372-K375 loop and Y376, as well as the C-terminus. MBD4^Δ/Δ^.12 is the most severely disrupted of the edited subclones and uracil glycosylase function is predicted to be disrupted.

### Chicken MBD4 Uracil Glycosylase Domain Deletions Lead to Increased SHM

**Figure 3:**
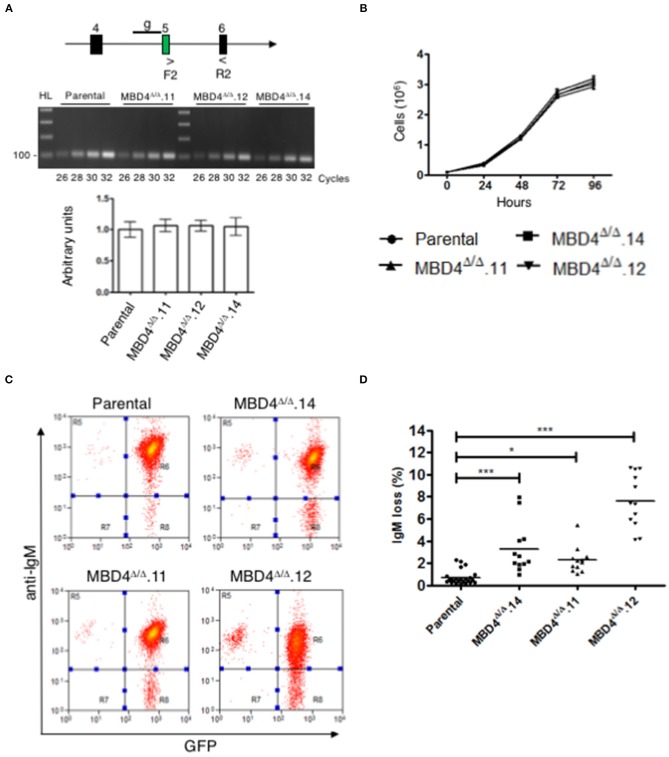
SHM is elevated in DT40 derived MBD4^Δ/Δ^ clones. **(A)** Schematic of exons 4, 5, and 6 in the Mbd4 gene and primers F2 and R2 used for RT-PCR analysis. gRNA sequence is indicated by a line (g) in early exon 5 (*upper panel*). Semi-quantitative RT-PCR analysis of Mbd4 exons 5–6 in parental and deleted clones (*middle panel*). QRT-PCR analysis of 18S RNA was used as a loading control (*lower panel*). 18S sample values were normalized to parental values which were set to 1. Samples were assayed in triplicate and were averaged, and SEMs are shown. **(B)** Cell proliferation assays. Cells (1 × 10^5^/ml) were grown in culture for 96 h and live cells were counted in triplicate every 24 h. The data shown is the average and is representative of 3 independent samples. **(C)** IgM fluorescence loss is analyzed in parental and MBD4^Δ/Δ^.14, MBD4^Δ/Δ^.11, and MBD4^Δ/Δ^.12 deletion clones by flow cytometry. Cells were FACS sorted at day 0 for IgM^+^GFP^+^ cells and re-analyzed after 28 days in culture. Flow cytometry analyses are representative of 24 parental- and 12 subclones from each of the MBD4^Δ/Δ^ deletion clones. **(D)** IgM fluorescence loss was quantitated for each parental and MBD4^Δ/Δ^ deletion subclone after 28 days in culture. Each data point is representative of a single sub-clone. Bars indicate the median in each data set. *P*-value *p < 0.05 and ***p < 0.0005 was analyzed using Kruskal-Wallis test.

Mbd4 gene expression was compared in DT40^c^ and Mbd4 deletion clones, MBD4^Δ/Δ^.14, MBD4^Δ/Δ^.11 and MBD4^Δ/Δ^.12, in semi-quantitative RT-PCR assays using primers spanning exons 5-6 (Figure 3A, upper panel). The 18S RNA loading control assessed in qRT-PCR analyses indicates that equal concentrations of cDNA were analyzed for each sample (Figure 3A, lower panel). Expression of Mbd4 transcripts was essentially identical in DT40^c^ as compared to the Mbd4 variant clones indicating that the DNA deletions did not impair Mbd4 steady state transcript levels. A direct assessment of MBD4 protein levels was not possible due to the lack of appropriate anti-MBD4 reagents.

SHM requires cell proliferation (34). To determine whether deletions in the Mbd4 gene influence cell proliferation, DT40^c^ cells and the MBD4^Δ/Δ^.14, MBD4^Δ/Δ^.11 and MBD4^Δ/Δ^.12 subclones were assessed. No differences were found in cell numbers over 96 h of cell growth between the DT40^c^ cells and the deletion subclones indicating that Mbd4 deletions have no impact on proliferation or viability (Figure 3B). Together, these studies indicate that DT40 cell growth and Mbd4 transcription are not affected by Mbd4 gene deletions.

We next asked whether deletions in the Mbd4 glycosylase domain impact SHM frequencies using the well-established criteria of IgM loss as a measure of SHM in DT40 cells (28). In this assay, loss of surface IgM is correlated with the frequency of deleterious mutations in the V(D)J exon of the IgH and the VJ exon of IgL. Thus, increased loss of IgM is indicative of greater SHM. Populations of GFP^+^*IgM*^+^ cells from DT40^c^, MBD4^Δ/Δ^.14, MBD4^Δ/Δ^.11 and MBD4^Δ/Δ^.12 lines were isolated by FACS at day 0 and subclones from each genotype were further isolated by limiting dilution. DT40^c^ (*n* = 24), MBD4^Δ/Δ^.14 (*n* = 12), MBD4^Δ/Δ^.11 (*n* = 12) and MBD4^Δ/Δ^.12 (*n* = 12) GFP^+^*IgM*^+^ subclones were cultured for 28 days and then analyzed for IgM expression levels by flow cytometry. Only cells expressing GFP, an indicator of AID expression, are included for IgM analyses. A representative histogram of IgM and GFP fluorescence for each genotype indicates that the percentage of IgM-GFP^+^ cells is greater for the Mbd4 deletion clones as compared to DT40^c^ cells when the same number of cells are analyzed (Figure 3C). Quantitative analyses indicate that MBD4^Δ/Δ^.14, MBD4^Δ/Δ^.11 and MBD4^Δ/Δ^.12 subclones were subject to significantly (*p* <0.0005) greater IgM loss as compared to DT40^c^ cells (Figure 3D). The greatest IgM loss was found in MBD4^Δ/Δ^.12 which also had the greatest predicted disruption to the MBD4 uracil glycosylase domain (Figures 2D, 3D). Previously, disrupting the UNG gene in DT40 was shown to lead to a very high rate of mutations in the VJ region at deaminated cytosine residues and the resulting uracils were not processed by uracil glycosylase activity (35). Further, UNG deficiency in mice and humans has been shown to lead to hypermutation at C/G bases (10, 36). This is consistent with our findings in chicken DT40 cells, in which disrupting MBD4 uracil glycosylase activity leads to increased mutation frequency.

Complementation studies designed to rescue MBD4 function are difficult to perform since overexpression of full length MBD4 leads to cell death in murine B cells (20). Consequently, we opted not to perform rescue studies here. We conclude that disruption of the catalytic amino acids in the MBD4 uracil glycosylase domain leads to a reduction in glycosylase activity and an increase in somatic hypermutation. This is shown by MBD4^Δ/Δ^.12 having the greatest predicted disruption to the uracil glycosylase domain structure and far higher levels of mutation. However, both MBD4^Δ/Δ^.11 and MBD4^Δ/Δ^.14 may retain some uracil glycosylase function as mutation frequency was lower than that found for MBD4^Δ/Δ^.12. Nevertheless, care must be taken interpreting the relationship between MBD4 structure and the magnitude of mutation frequency as clone to clone variation cannot be completely eliminated.

### Mutations in the IgL of the Mbd4 Deletion Clones Are Predominantly at G:C Nucleotides

**Figure 4:**
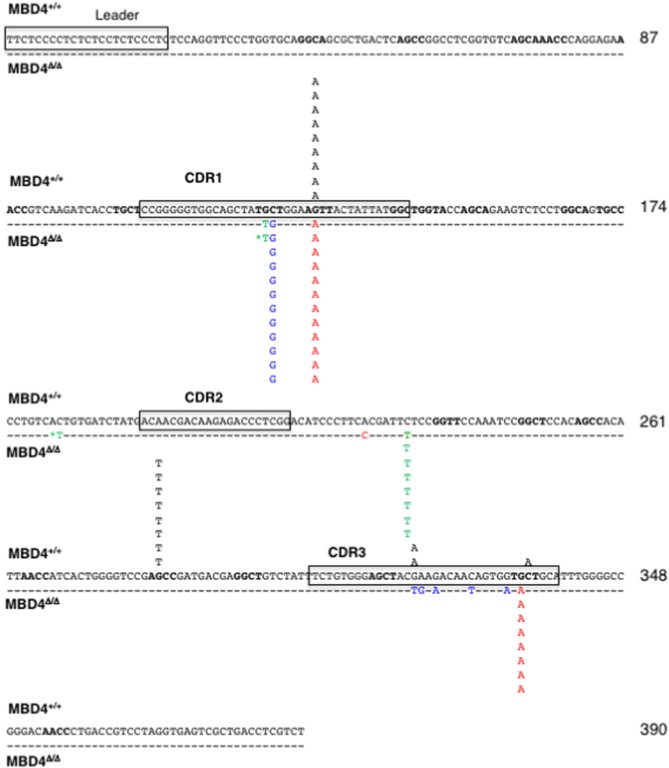
SHMs occur largely at G/C residues in the IgL gene of MBD4^Δ/Δ^ subclones. The leader and CDR1, CDR2 and CDR3 motifs in the reference sequence are boxed. Mutations are from the parental (*n* = 11, black; above the reference sequence) and MBD4^Δ/Δ^.11 (*n* = 8, green), MBD4^Δ/Δ^.12 (*n* = 12, blue), and MBD4^Δ/Δ^.14 (*n* = 12, red) deletion clones, below the reference sequence. The asterisk indicates that the mutated nucleotide sequence was mixed with the parental sequence at that position. AID hotspot motifs (RGYW, WRCY) are highlighted in bold. Nucleotide numbering is indicated at the end of each line.

To analyze the impact of Mbd4 deletion on SHM the rearranged VJ regions of IgL were PCR amplified and sequenced from DT40^c^ and Mbd4 deletion subclones isolated in the IgM loss study (Figures 3C,D). This approach to mutation analysis will detect substitutions, deletions and insertions that are in the majority of cells of a subclone and not rare mutations that may have occurred late in the culture period. Nucleotide changes were found in the 0.39 kb amplicon between the V leader and the 5′ end of the J-C intron and focused to AID hotspots (RGYW, WRCY) (Figure 4). The number of mutations ranged from 0-2 in DT40^c^, 1–2 in MBD4^Δ/Δ^.14 and MBD4^Δ/Δ^.11 and 1–4 in MBD4^Δ/Δ^.12 subclones. Many of the mutations are located in the same positions as would be expected after clonal expansion. Nevertheless, a number of clones have acquired unique mutations in addition to the dominant mutation, indicating that SHM was operational in those clones (Supplementary Figures 1–4). No insertions or deletions were detected. All mutations were sequenced on both the forward and reverse strands, were unambiguous and were present in the great majority of cells as no background was evident. Two mutated nucleotides noted by asterisks were present in equal quantity with control sequence indicating that the PCR products were mixed at that position and that the mutations occurred in a subset of cells in the subclone (Figure 4). Mutations were predominantly G:C transitions and transversions for all subclones tested as previously noted for DT40 B cells (Figure 4) (28). The clonal nature of mutations in the VJ exon precludes calculation of mutation frequency. Therefore, we infer that SHM frequency is increased in the Mbd4 deletion subclones based on the increase of IgM loss.

These results indicate that both MBD4 and UNG glycosylases are involved in recognizing and removing AID-induced uracils. Earlier analyses demonstrated that MBD4 interacts with MLH1 in mice and therefore might be involved in regulating the rectification of T:G and U:G mismatches via MMR (21). Although earlier genetic studies suggested that MLH1 does not function in SHM (37, 38) more recent work has revealed the importance of MLH1 for SHM in mouse (14). However, the vast majority of mutations in DT40 cells are G:C transitions and transversions (28). The diminished frequency of A:T mutations implies that error-prone synthesis by Pol η in the MMR pathway is negligible in these cells. Therefore, the exact mechanism by which MBD4 influences SHM in mice and humans remains to be determined. It should be noted that the DT40 IgM loss assay relies on complementation with overexpressed AID which may in turn influence the efficiency of uracil recognition by MBD4. Confirmation of the role of MBD4 in SHM requires analysis in a setting with endogenous levels of AID.

## Data Availability Statement

All datasets generated for this study are included in the article/Supplementary Material.

## Author Contributions

The protein comparison of annotated Mbd4 species (Figure 1A) and multiple protein sequence alignments (Figure 1B), the DT40 culture and CRISPR/Cas9 editing experiments and analyses (Figures 2A,B) were designed by AK and RC and performed by RC. JC and AK designed and JC performed the 3D structure analysis and alignment (Figures 1C, 2C). All cell proliferation and cDNA/DNA analyses (Figures 3A,B) as well as the flow cytometry for the IgM loss assay (Figures 3C,D) were designed by AK and RC and carried out by RC. Mutation analysis (Figure 4) was designed by AK and generated by JC. The manuscript was written by RC and AK. AK critically revised the paper and all authors approved the final version of the manuscript.

### Conflict of Interest

The authors declare that the research was conducted in the absence of any commercial or financial relationships that could be construed as a potential conflict of interest.

## Abbreviations

AID: Activation induced deaminase
AP: Apurinic/pyrimidinic
CSR: Class switch recombination
BER: Base excision repair
FACS: Fluorescence-activated cell sorting
gRNA: guide RNA
HhH: Helix hairpin helix
MBD4: Methyl binding domain protein 4
MLH1: MutL homolog 1
MMR: Mismatch repair
MSI: miscrosatellite instability
RNP: Ribonuclear protein
SHM: Somatic hypermutation
SMUG1: Single-strand selective monofunctional uracil DNA glycosylase
TDG: Thymine DNA glycosylase
UNG: Uracil DNA glycosylase
WT: wild type.
