# Supplementary material for: Chicken MBD4 Regulates Immunoglobulin Diversification by Somatic Hypermutation
Source: Front Immunol. 2019 Nov 1;10:2540. doi: 10.3389/fimmu.2019.02540 (PMC6838969; doi:10.3389/fimmu.2019.02540)
Supplement: Supplementary file 1 [file Presentation_1.pdf]

```
MBD4+/+
                                Leader
TTCTCCCCTCTCTCCTCTCCCTCTCCAGGTTCCCTGGTGCAGGCAGCGCTGACTCAGCCGGCCTCGGTGTCAGCAAACCCAGGAGAA
---------------------------------------------------------------------------------------
MBD4Δ/Δ


MBD4+/+
                                CDR1
ACCGTCAAGATCACCTGCTCCGGGGGTGGCAGCTATGCTGGAAGTTACTATTATGGCTGGTACCAGCAGAAGTCTCCTGGCAGTGCC
C11.1-------------------------------T--------------------------------------------------
MBD4Δ/Δ C11.3                      *T


MBD4+/+
                                CDR2
CCTGTCACTGTGATCTATGACAACGACAAGAGACCCTCGGACATCCCTTCACGATTCTCCGGTTCCAAATCCGGCTCCACAGCCACA
C11.1---------------------------------------------------T------------------------------
MBD4Δ/Δ C11.3                                               T
C11.4 *T                                                T
C11.5                                                   T
C11.7                                                   T
C11.8                                                   T
C11.10                                                  T
C11.12                                                  T

MBD4+/+
                                                            CDR3
TTAACCATCACTGGGGTCCGAGCCGATGACGAGGCTGTCTATTTCTGTGGGAGCTACGAAGACAACAGTGGTGCTGCATTTGGGGCC
---------------------------------------------------------------------------------------
MBD4Δ/Δ


MBD4+/+
GGGACAACCCTGACCGTCCTAGGTGAGTCGCTGACCTCGTCT
------------------------------------------
MBD4Δ/Δ
```

Suppl. Figure 1. SHM in the IgL gene of MBD4$^{\Delta/\Delta}$.11 subclones. The leader and CDR1, CDR2 and CDR3 motifs in the reference sequence are boxed. Mutations are from MBD4$^{\Delta/\Delta}$.11 (n=8, green). AID hotspot motifs (RGYW, WRCY) are highlighted in bold.

```
MBD4+/+
                    Leader
TTCTCCCCTCTCTCCTCTCCCTCTCCAGGTTCCCTGGTGCAGGCAGCGCTGACTCAGCCGGCCTCGGTGTCAGCAAACCCAGGAGAA
---------------------------------------------------------------------------------------
MBD4Δ/Δ


MBD4+/+
                          CDR1
ACCGTCAAGATCACCTGCTCCGGGGGTGGCAGCTATGCTGGAAGTTACTATTATGGCTGGTACCAGCAGAAGTCTCCTGGCAGTGCC
C12.1--------------------------------G-------------------------------------------------
MBD4Δ/Δ
C12.2                                G
C12.3                                G
C12.4                                G
C12.5                                G
C12.6                                G
C12.7                                G
C12.8                                G
C12.9                                G
C12.10                               G
C12.11                               G
C12.12                               G

MBD4+/+
                          CDR2
CCTGTCACTGTGATCTATGACAACGACAAGAGACCCTCGGACATCCCTTCACGATTCTCCGGTTCCAAATCCGGCTCCACAGCCACA
---------------------------------------------------------------------------------------
MBD4Δ/Δ


MBD4+/+
                                                    CDR3
TTAACCATCACTGGGGTCCGAGCCGATGACGAGGCTGTCTATTTCTGTGGGAGCTACGAAGACAACAGTGGTGCTGCATTTGGGGCC
C12.1----------------------------------------------------TG-A--------------------------
MBD4Δ/Δ
C12.9                                                            T    A


MBD4+/+
GGGACAACCCTGACCGTCCTAGGTGAGTCGCTGACCTCGTCT
------------------------------------------
MBD4Δ/Δ
```

Suppl. Figure 2. SHMs occur in the IgL gene of MBD4$^{\Delta/\Delta}$.12 subclones. The leader and CDR1, CDR2 and CDR3 motifs in the reference sequence are boxed. Mutations are from MBD4$^{\Delta/\Delta}$.12 (n=12, blue) below the reference sequence. AID hotspot motifs (RGYW, WRCY) are highlighted in bold.

$MBD4^{+/+}$

Leader

```
TTCTCCCCTCTCTCCTCTCCCTCTCCAGGTTCCCTGGTGCAGGCAGCGCTGACTCAGCCGGCCTCGGTGTCAGCAAACCCAGGAGAA
---------------------------------------------------------------------------------------
```

$MBD4^{\Delta/\Delta}$

$MBD4^{+/+}$

CDR1

```
ACCGTCAAGATCACCTGCTCCGGGGGTGGCAGCTATGCTGGAAGTTACTATTATGGCTGGTACCAGCAGAAGTCTCCTGGCAGTGCC
C14.1--------------------------------------A-------------------------------------------
C14.2                                      A
C14.3                                      A
C14.4                                      A
C14.5                                      A
C14.6                                      A
C14.7                                      A
C14.8                                      A
C14.9                                      A
C14.10                                     A
C14.11                                     A
C14.12                                     A
```

$MBD4^{\Delta/\Delta}$

$MBD4^{+/+}$

CDR2

```
CCTGTCACTGTGATCTATGACAACGACAAGAGACCCTCGGACATCCCTTCACGATTCTCCGGTTCCAAATCCGGCTCCACAGCCACA
C14.10--------------------------------------------C------------------------------------
```

$MBD4^{\Delta/\Delta}$

$MBD4^{+/+}$

CDR3

```
TTAACCATCACTGGGGTCCGAGCCGATGACGAGGCTGTCTATTTCTGTGGGAGCTACGAAGACAACAGTGGTGCTGCATTTGGGGCC
C14.1-------------------------------------------------------------------A--------------
C14.2                                                                   A
C14.4                                                                   A
C14.6                                                                   A
C14.8                                                                   A
C14.9                                                                   A
C14.11                                                                  A
C14.12                                                                  A
```

$MBD4^{\Delta/\Delta}$

$MBD4^{+/+}$

```
GGGACAACCCTGACCGTCCTAGGTGAGTCGCTGACCTCGTCT
------------------------------------------
```

$MBD4^{\Delta/\Delta}$

Suppl. Figure 3. SHMs occur largely at G/C residues in the IgL gene of MBD4$^{\Delta/\Delta}$ **subclones.**
The leader and CDR1, CDR2 and CDR3 motifs in the reference sequence are boxed. Mutations are from MBD4$^{\Delta/\Delta}$.14 (n=12, red) deletion clones, below the reference sequence. AID hotspot motifs (RGYW, WRCY) are highlighted in bold.

MBD4+/+

Leader

```
TTCTCCCCTCTCTCCTCTCCCTCTCCAGGTTCCCTGGTGCAGGCAGCGCTGACTCAGCCGGCCTCGGTGTCAGCAAACCCAGGAGAA
---------------------------------------------------------------------------------------
```

MBD4Δ/Δ

MBD4+/+

CDR1

```
WT.12                                      A
WT.11                                      A
WT.9                                       A
WT.8                                       A
WT.6                                       A
WT.5                                       A
WT.4                                       A
WT.3                                       A
WT.2                                       A
WT.1                                       A
ACCGTCAAGATCACCTGCTCCGGGGGTGGCAGCTATGCTGGAAGTTACTATTATGGCTGGTACCAGCAGAAGTCTCCTGGCAGTGCC
---------------------------------------------------------------------------------------
```

MBD4Δ/Δ

MBD4+/+

CDR2

```
CCTGTCACTGTGATCTATGACAACGACAAGAGACCCTCGGACATCCCTTCACGATTCTCCGGTTCCAAATCCGGCTCCACAGCCACA
---------------------------------------------------------------------------------------
```

MBD4Δ/Δ

MBD4+/+

CDR3

```
WT.12                T
WT.11                T
WT.9                 T                                   A
WT.8                                                        C
WT.6                 T                                   A
WT.5                 T                                   A
WT.4                 T
WT.3                 T
WT.2                 T                                   A
WT.1                 T
TTAACCATCACTGGGGTCCGAGCCGATGACGAGGCTGTCTATTTCTGTGGGAGCTACGAAGACAACAGTGGTGCTGCATTTGGGGCC
---------------------------------------------------------------------------------------
```

MBD4Δ/Δ

MBD4+/+

```
GGGACAACCCTGACCGTCCTAGGTGAGTCGCTGACCTCGTCT
------------------------------------------
```

MBD4Δ/Δ

Suppl. Figure 4. SHMs occur in the IgL gene of MBD4+/+ subclones. The leader and CDR1, CDR2 and CDR3 motifs in the reference sequence are boxed. Mutations are from the parental (n=11, black; above the reference sequence). AID hotspot motifs (RGYW, WRCY) are highlighted in bold.
